# Formation of disk-like micelles of triblock copolymers in frustrating solvents†

**DOI:** 10.1039/c9ra01145e

**Published:** 2019-03-25

**Authors:** Hongyan Zhu, Yan Cui, Jie Wang, Huibin Qiu

**Affiliations:** Shanghai Institute of Applied Physics, Chinese Academy of Sciences Shanghai 201800 China wangjie@sinap.ac.cn; University of Chinese Academy of Sciences Beijing 100049 China; School of Chemistry and Chemical Engineering, State Key Lab of Metal Matrix Composites, Shanghai Jiao Tong University Shanghai 200240 China hbqiu@sjtu.edu.cn; Shanghai Advanced Research Institute, Zhangjiang Lab, Chinese Academy of Sciences Shanghai 201204 China

## Abstract

Coil–coil block copolymers rarely self-assemble into flat low-curvature micelles due to the lack of proper interchain association. Here, we report a facile route to prepare disk-like micelles through the self-assembly of amphiphilic polystyrene-*b*-polybutadiene-*b*-poly(2-vinylpyridine) triblock copolymers in a mixture of acetone and cyclohexane, which shows distinct selectivity towards the PS, PB and P2VP blocks. Subtle solvation/aggregation of these blocks in this frustrating solvent system provides access to low-curvature micellar structures, and thus favors the formation of uniform disk-like micelles. Further variation of the volume ratio of the mixed solvents also leads to the emergence of other interesting morphologies, including disk arrays, disk clusters and perforated disk-like micelles. This work provides a complementary insight into the solution self-assembly of block copolymers in a view of selective solvents and demonstrates a distinctive pathway to unconventional micellar nanostructures through the use of complex solvent systems.

## Abstract 2

Self-assembly of amphiphilic triblock copolymers in a frustrating solvent system leads to the formation of various low-curvature micellar structures.

## Graphical abstract

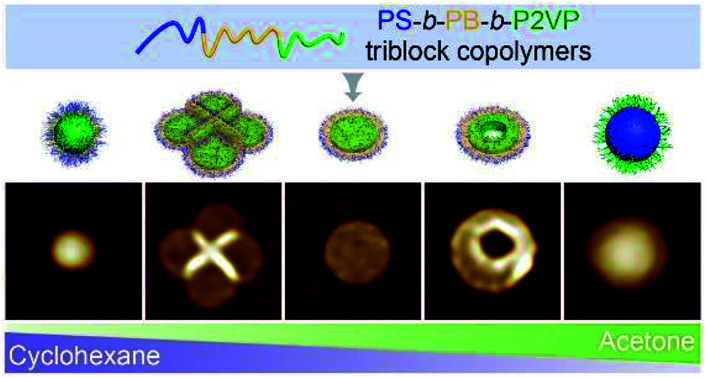

## Introduction

Amphiphilic block copolymers (BCPs) have been demonstrated to be a family of versatile and promising materials to construct a diverse array of nanostructures.^1–3^ In the past few decades, numerous studies have been focused on the fabrication and characteristics of various micellar morphologies.^1–4^ In solution, these were mainly achieved by adjusting the chemistry and composition of BCPs, solvent selectivity, and external conditions,^2,5^ such as temperature,^6,7^ pH^7,8^ and salts.^8–10^ Besides, complex micellar structures, including toroids,^11,12^ janus^4^ and multicompartment micelles,^13–15^ can be realized by more exquisite self-assembly of diblock and triblock copolymers.

Among these morphologies, disk-like micelles were relatively rarely observed and so far were generally fabricated by the use of (i) rod–coil BCPs with a conjugated block;^16–18^ (ii) crystalline–coil BCPs with a crystallization block (through crystallization-driven self-assembly)^19–21^ and (iii) polypeptide-derived BCPs.^22–24^ The presence of a rigid core-forming block appeared to be essential for the formation of flat (low-curvature) self-assembly nanostructures. Due to the lack of requisite stiffness, coil–coil BCPs were considered to be unfavourable for the formation of disk-like micelles, although a limited number of exceptions were found previously.^25–29^ These normally involved the introduction of particular counterions in the self-assembly of coil–coil BCPs with an ionic corona-forming block by adjusting the interfacial energy,^30–32^ or chemical modification of one chain block to finely tune the incompatibility toward the selective solvents.^33–35^ Consequently, it has remained a challenge to assemble coil–coil BCPs into discotic micellar morphologies *via* a simple, efficient and tunable route.

Herein, we developed a facile strategy to prepare disk-like micelles through the self-assembly of amphiphilic polystyrene-*b*-polybutadiene-*b*-poly(2-vinylpyridine) (PS-*b*-PB-*b*-P2VP) triblock copolymers in a frustrating solvent system that consists acetone and cyclohexane. This also enabled the formation of disk arrays, disk clusters and perforated disk-like micelles simply by tuning the volume ratio of acetone and cyclohexane, which provided further insights into the formation of low-curvature micelles and the sophisticate association between amphiphilic BCPs and solvents.

## Experimental

### Materials

*sec*-Butyllithium (*sec*-BuLi, 1.3 M in hexane, Aldrich), *n*-butyllithium (*n*-BuLi, 1.6 M in hexane, Aldrich) and trioctylaluminum (TOA, 25 wt% solution in hexane, Aldrich) were used without further purification. All monomers and reagents were purified under reduced pressure distillation. Typically, styrene was dried over CaH_2_ overnight on the N_2_ flow and then degassed with three freeze–pump–thaw cycles. Next, styrene was distilled into another flask. Subsequent, an appropriate amount of TOA was added into styrene in an inert argon atmosphere glovebox and stirred for *ca.* 30 min. Finally, styrene was distilled into a clean flask under reduced pressure and stored in −30 °C in glovebox before use. 1,3-Butadiene (*ca.* 13% in tetrahydrofuran (THF)) solution was dried over CaH_2_ for 30 min on the N_2_ flow at −10 °C (ice/salt bath).^36^ Then it was degassed with three freeze–pump–thaw cycles and distilled into another flask. An appropriate amount of TOA was added into 1,3-butadiene in the glovebox and stirred for *ca.* 30 min at −10 °C. Finally, 1,3-butadiene was distilled into a clean flask under reduced pressure and stored in −30 °C in glovebox before use. 2-Vinylpyridine and diphenylethylene (DPE) was purified as styrene. THF was refluxed over Na in the presence of benzophenone under N_2_ atmosphere, until a bright deep purple color appeared. And then a drop of DPE was added into THF, subsequent *n*-BuLi was added drop-wise to the solvent until the color became deep red color, which indicated THF was free from impurities.^36^ Finally, THF was distilled into a clean flask and stored in −30 °C in glovebox before use.

### Synthesis of PS-*b*-PB-*b*-P2VP triblock copolymers

PS-*b*-PB-*b*-P2VP block copolymers were synthesized *via* living anionic polymerization in THF using *sec*-BuLi as initiator in an inert argon atmosphere glovebox. In a typical synthesis process, *n*-BuLi was added into the purified THF under stirring and aged at room temperature (RT ≈ 25 °C) overnight to induced lithium alkoxides into the reaction medium.^37^ On the next day, a small amount of styrene was introduced into a THF solution and cooled to −80 °C (liquid nitrogen/ethanol bath). And then a calculated amount of *sec*-BuLi was added as initiator under vigorous stirring to give the orange solution. After 30 min, an aliquot of the precursor was taken for gel permeation chromatography (GPC) analysis. Then the second monomer, 1,3-butadiene, was added under vigorous stirring, causing a change of color from orange to colourless, and polymerized at −30 °C for 6 h. After the reaction was completed, an aliquot of the diblock precursor was taken for GPC analysis. DPE was added into the reaction mixture in 10-fold excess. The red color gradually developed within minutes. After, the solution was cooled to −80 °C, and 2-vinylpyridine was added. The solution became deep red and was subjected to vigorous stirring for 1 h until completion of the reaction. Finally, the polymerization was terminated by *p-tert*-butylphenol.

### Solution self-assembly of PS-*b*-PB-*b*-P2VP

The block copolymers were firstly dissolved in THF (20 mg mL^−1^) by stirring at room temperature (RT ≈ 25 °C). Mixtures of acetone and cyclohexane, or isopropanol (IPA) and cyclohexane, with various volume ratios were prepared in advance. Typically, 40 μL of the unimer solution of PS-*b*-PB-*b*-P2VP in THF was quickly added into 1 mL of mixed solvents. The mixture was vigorously shaken with a vortex mixer for ∼5 s and then allow to age at RT for at least 1 day. The final concentration of the solution was about 0.769 mg mL^−1^.

### Characterizations

Proton nuclear magnetic resonance (^1^H NMR) were recorded on a Brucker AVANCE III HD 500 MHz spectrometer. All spectra were obtained using deuterated chloroform (CDCl_3_) as the solvent. The ^1^H chemical shifts were determined using the residual signals of the deuterated solvents (7.26) as the internal standard (see ESI† for analytical details).

GPC was carried out on a Viscotek TDAMax Multidetector Gel Permeation Chromatograph. The detectors and the chromatography were controlled in an oven (25 °C). THF (Fisher) was used as the eluent, with a flow rate of 1.0 mL min^−1^. Samples were dissolved in the eluent (2 mg mL^−1^) and filtered with a filter (Titan, polytetrafluoroethylene membrane with 0.22 μm pore size) before analysis. Calibration was conducted using TDS-PS-NB-polystyrene standards (PS105K and PS245K) from Viscotek. To determine the molar mass of the block copolymers, aliquots of the first block were taken and the absolute molar mass of the first block was determined by GPC. The absolute molecular weights of the other two blocks were then determined by combining the molecular weight *M*_n_ of the first block from GPC measurements with the block ratio of the triblock copolymer which was obtained by integration of the ^1^H NMR spectrum (see ESI† for analytical details).

Atomic force microscopy (AFM) experiments were performed using a Bruker Dimension ICON scanning probe microscope equipped with a ScanAsyst-HR scanning accessory. Imaging was scanned by a ScanAsyst-Air prober (tip radius 5 nm) under ScanAsyst mode in the air at room temperature. Images were analyzed by NanoScope Analysis, an open source software program for AFM images. Samples were prepared by placing one drop (*ca.* 20 μL) of the solution onto a piece of freshly peeled mica.

Transmission electron microscopy (TEM) experiments were performed using a Tecnai G2 Spirit transmission electron microscope operating at 120 kV and equipped with a 4k × 4k Eagle CCD. Samples were prepared by placing one drop (*ca.* 10 μL) of the solution on a carbon-coated copper grid resting on a piece of filter paper to remove excess solvent. No staining of the samples was necessary.

## Results and discussion

Three amphiphilic triblock copolymers with a hydrophilic poly(2-vinylpyridine) (P2VP) block and two hydrophobic polystyrene (PS) and polybutadiene (PB) blocks, namely PS_580_-*b*-PB_660_-*b*-P2VP_920_, PS_370_-*b*-PB_230_-*b*-P2VP_350_, and PS_260_-*b*-PB_170_-*b*-P2VP_300_ (the subscripts represent the number-average degree of polymerization), were prepared through living anionic polymerization and characterized by NMR spectrum and GPC (Fig. S1–S6†). The characteristics of the synthesized triblock copolymers are summarized in Table 1.

**Table 1:** Characteristics of the synthesized PS-*b*-PB-*b*-P2VP triblock copolymers

Polymer	PDI	*M*_n_ (g mol^−1^)	Block ratio (P2VP/PS)	Block ratio (P2VP/PB)
PS_260_-*b*-PB_170_-*b*-P2VP_300_	1.14	67 800	∼1.2	∼1.8
PS_370_-*b*-PB_230_-*b*-P2VP_350_	1.22	87 800	∼0.9	∼1.5
PS_580_-*b*-PB_660_-*b*-P2VP_920_	1.25	192 800	∼1.6	∼1.4

**Fig. 1:**
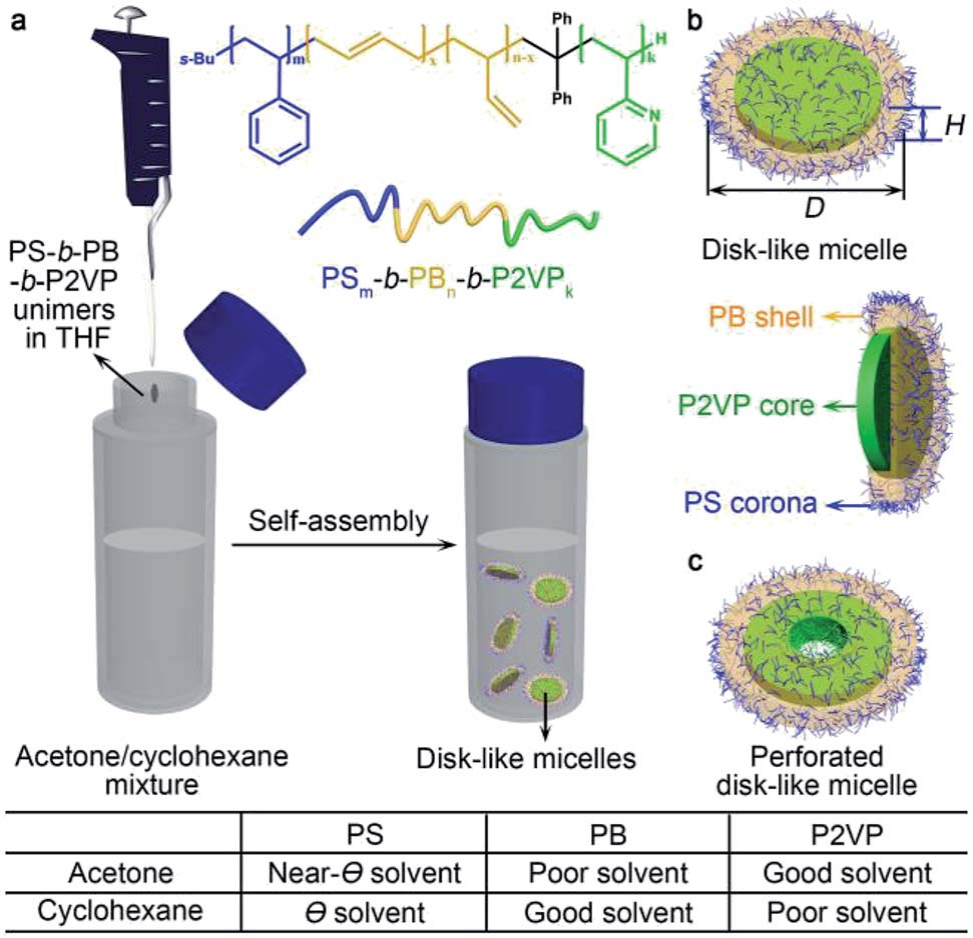
Schematic representations for (a) the formation process of disk-like micelles in a mixture of acetone and cyclohexane, and (b and c) possible structures of disk-like and perforated disk-like micelles, respectively.

In a typical self-assembly experiment, PS-*b*-PB-*b*-P2VP was dissolved in THF to make a concentrated unimer solution (20 mg mL^−1^). An aliquot (40 μL) of the unimer solution was quickly injected into a mixture of acetone and cyclohexane. In this case, acetone serves as a near-*Θ* solvent for PS, but poor solvent for PB and good solvent for P2VP,^13^ while cyclohexane is a *Θ* solvent for PS,^38^ good solvent for PB, but poor solvent for P2VP (Fig. 1). The solubility parameters of the polymers (PS, PB, and P2VP) and solvents, and the Flory–Huggins parameters (*χ*) (polymer–solvent interaction strengths) were listed in Tables S1 and S2,† respectively.

**Fig. 2:**
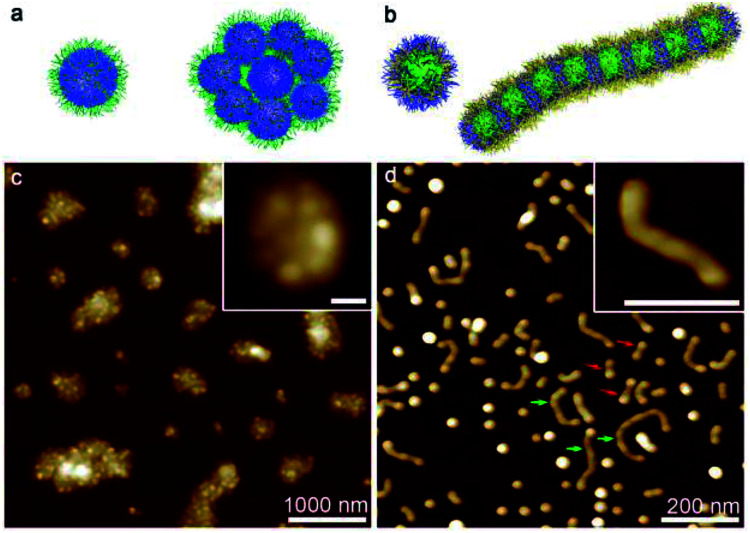
AFM height images and schematic representations of nanostructures formed by the self-assembly of PS_580_-*b*-PB_660_-*b*-P2VP_920_ in (a and c) acetone and (b and d) cyclohexane. The scale bars correspond to 100 nm in the inset images.

In consideration of the distinct solubility feature of the PS, PB and P2VP blocks, we firstly explored the self-assembly of PS_580_-*b*-PB_660_-*b*-P2VP_920_ in pure acetone and cyclohexane, respectively. In acetone, the solution appeared as a white suspension, and AFM images indicated the presence of random aggregates of spherical micelles (Fig. 2a and c). According to the solubility preference, these spherical micelles were supposed to possess a PB core and a P2VP corona, with the PS block partially swelled (acetone is a near-*Θ* solvent for PS) and deposited at the core–corona interface. The association of the less solvable PS block may thus render the coagulation of the spherical micelles into irregular clusters. In cyclohexane, AFM images showed the formation of a mixture of spherical micelles and worm-like micelles (Fig. 2b and d). While the P2VP block aggregated into a core and the PB block formed the corona of a spherical micelle, the solvation of the PS block in such a *Θ* solvent was rather frustrated (the polymer chain normally forms coil but does not fully extend in a *Θ* solvent).^39^ It presumably caused the coalescence of the spherical micelles and consequently the emergence of the elongated micellar structures.^13,40^ This was also indicated by the dumbbell-like shape of the short worm-like micelles (denoted by red arrows) and the beam pod-like feature of the longer species (denoted by green arrows) (Fig. 2d). It seems that the PS-*b*-PB-*b*-P2VP triblock copolymer self-assembled in an unconventional manner in both acetone and cyclohexane, majorly as a consequence that PS swells but not actually dissolves in these solvents. However, the solubility of PS increases in a mixture of acetone and cyclohexane due to the cosolvency effect.^41–43^ Thus, it would be interesting to reveal the self-assembly behaviour of the PS-*b*-PB-*b*-P2VP triblock copolymer in various acetone/cyclohexane mixtures.

To this end, acetone and cyclohexane were mixed in different volume ratios and used for the self-assembly experiments. In the cases of relatively low acetone/cyclohexane ratios (≤30/70), PS_580_-*b*-PB_660_-*b*-P2VP_920_ predominantly formed uniform spherical micelles (Fig. S7b–d†). With an increasing in the amount of acetone (acetone/cyclohexane = 40/60), the spherical micelles were slightly deformed and concave dimples were formed on the surface (Fig. S7e†). This was presumably due to the swell of the P2VP core and the compression of the PB corona in a heavier acetone atmosphere.

**Fig. 3:**
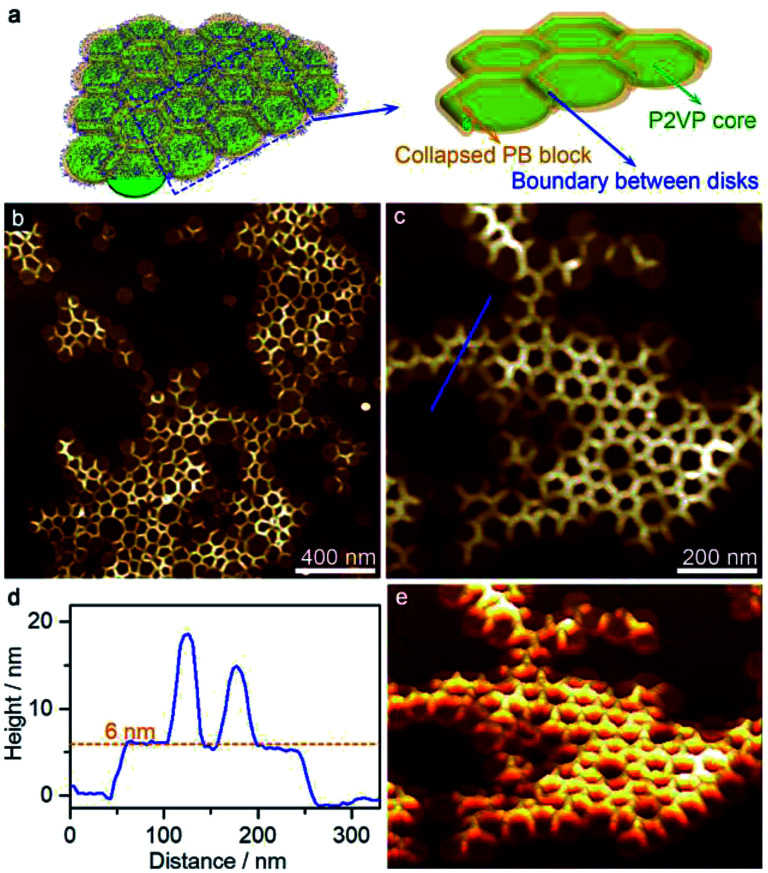
(a) Schematic representation, (b and c) AFM height images, (d) height profiles, and (e) 3D images of disk arrays formed by the self-assembly of PS_580_-*b*-PB_660_-*b*-P2VP_920_ in a 45/55 acetone/cyclohexane mixture.

A further increase of acetone (acetone/cyclohexane = 45/55) led to the formation of disk arrays with the coexistence of discrete disks (Fig. 3). AFM height and 3D images showed that the disk arrays consisted of lateral connected disks with height of ∼6 nm. The boundary (height ∼ 15 nm) where the disks connected with each other was significantly higher than the disk center, forming the “mountains”, and the area of the “basins” was slightly smaller than that of the individual disks, indicating a considerable collision of the disks. This strongly indicated that the disk arrays formed directly in solution, rather than as a consequence of a dry effect where loosely packed aggregates would be most possible.

**Fig. 4:**
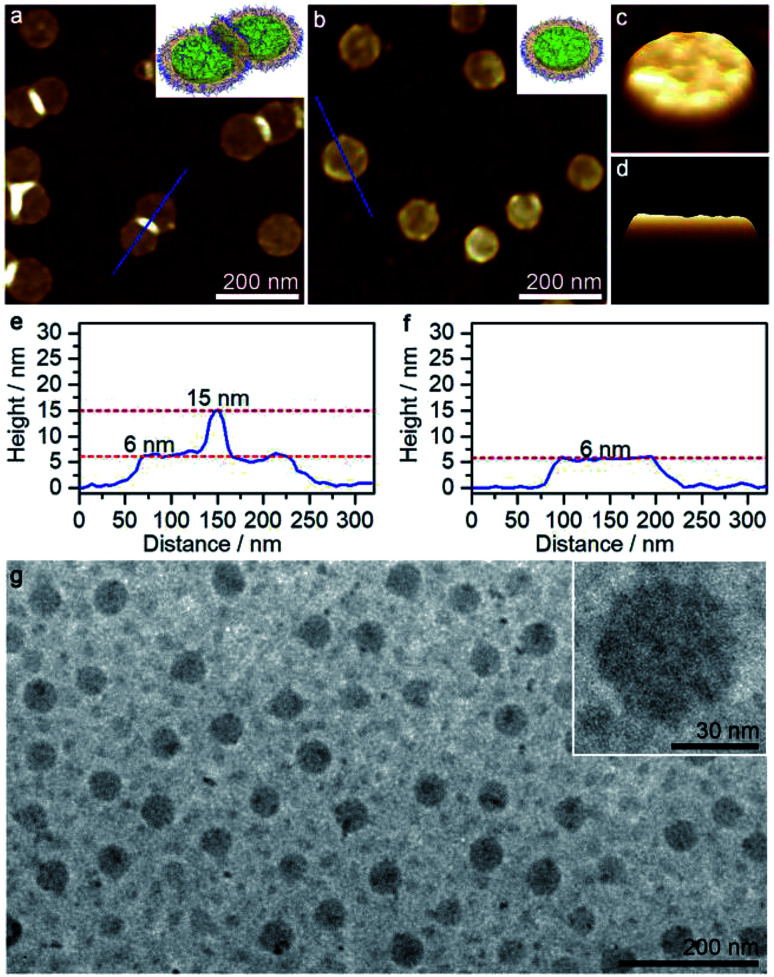
AFM height images and height profiles of disk structures formed by the self-assembly of PS_580_-*b*-PB_660_-*b*-P2VP_920_ in (a and e) 55/45 and (b and f) 60/40 acetone/cyclohexane mixtures, respectively. (c and d) AFM 3D images of a disk-like micelle corresponding to the sample shown in (b). (g) TEM images of disk-like micelles formed in the 60/40 acetone/cyclohexane mixture.

The aforementioned large disk arrays disappeared in the 50/50 acetone/cyclohexane mixture, in which relatively small clusters were detected instead (Fig. S7g†). The disks were again laterally linked together, with aggregation number varying from 2 to 15. At acetone/cyclohexane = 55/45, the solution mainly produced disk trimers and dimers, and a large portion of individual disks (Fig. 4a and e). Morphologically pure disk-like micelles with diameter of ∼100 nm (estimated standard deviations *σ* = 23 nm by AFM) and height of ∼6 nm were observed at acetone/cyclohexane = 60/40 (Fig. 4b and f). AFM 3D images showed that the disk-like micelles possessed a slight rough surface (Fig. 4c and d). Moreover, TEM images further confirmed the presence of disk-like micelles (Fig. 4g), although the diameter appeared to be much smaller, probably due to the invisible corona under the electron beam.

**Fig. 5:**
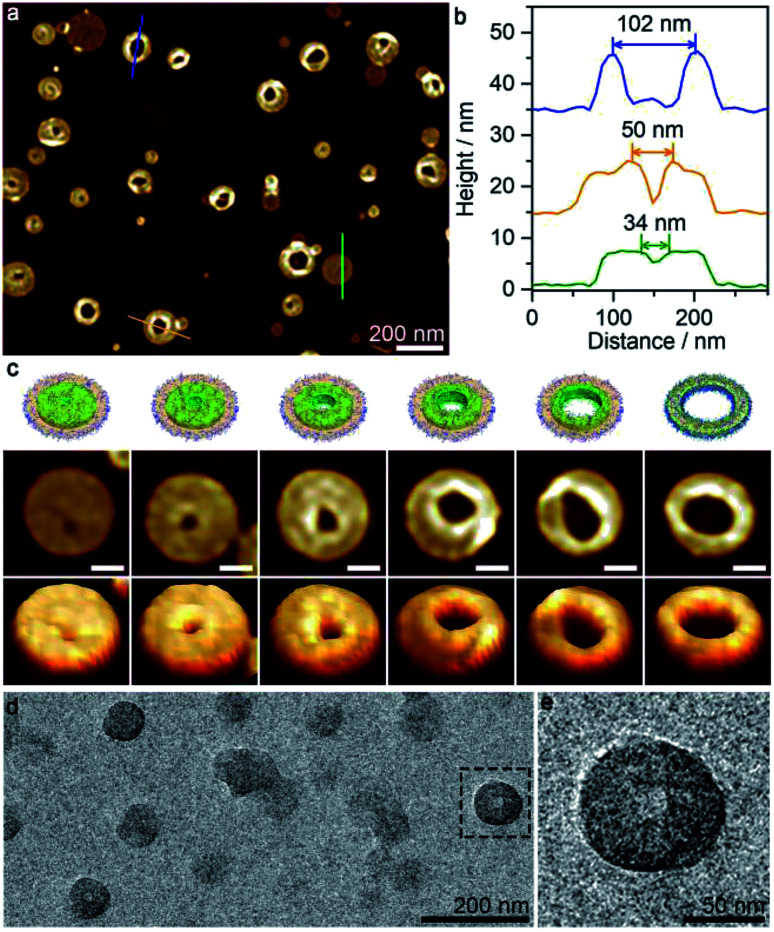
(a) AFM height images and (b) height profiles of perforated disk-like micelles formed by the self-assembly of PS_580_-*b*-PB_660_-*b*-P2VP_920_ at acetone/cyclohexane = 65/35. (c) Schematic representations, AFM height images and 3D images showing the perforated disk-like micelles with various hole sizes. The scale bars correspond to 50 nm. (d and e) TEM images of perforated disk-like micelles.

Interestingly, perforated disk-like micelles emerged when the acetone/cyclohexane ratio was increased to 65/35 (Fig. 5 and S7h†). The size of holes varied in a wide range from 5 nm to 100 nm, and the relatively large holes generally adopted irregular shapes (Fig. 5b and c). Notably, the periphery of the holes (height > 10 nm) was obviously higher than the intact disk areas (height ∼ 8 nm). It appeared that the perforation occurred after the formation of the disk-like micelles through a process similar to piercing a paper. The perforated disk-like micelles were also detected by TEM images (Fig. 5d and e), excluding a drying effect (for AFM, samples were deposited on mica, a relatively hydrophilic surface, while for TEM, samples were deposited on carbon film, a relatively hydrophobic surface).

For acetone/cyclohexane ≥70/30, where acetone became dominant, PS_580_-*b*-PB_660_-*b*-P2VP_920_ majorly self-assembled into spherical micelles (Fig. S7i–k†).

It was apparent that the combination of acetone and cyclohexane sophisticated the self-assembly of PS_580_-*b*-PB_660_-*b*-P2VP_920_. Although the exact mechanism was not clear yet, it was postulated that: (i) due to the cosolvency effect, the PS block dissolves well in the acetone/cyclohexane mixtures with moderated volume ratios and thus essentially/additionally stabilizes the resulting micellar structures; (ii) with an increase in the acetone fraction, the PB corona gradually contracts and aggregates, while the P2VP core swells, leading to a decrease in the corona/core volume ratio, and consequently the transition from high-curvature spherical micelles to low-curvature discotic micelles; (iii) at acetone/cyclohexane of 45/55–55/45, the PB block partially collapses but yet retains as a soft/adhesive layer along the interface of the PS corona and the P2VP core, and thus causes the tethering and collision of the disk-like micelles; (iv) the further solvation and stretching of the P2VP core under a higher acetone/cyclohexane ratio destabilizes the disk structures and thus perforation occurs (compared to the intact disk micelles that formed at acetone/cyclohexane of 60/40, an increase of acetone might cause the shrinkage of the PB corona and a swelling of the hydrophilic P2VP core, resulting in an eruption of the P2VP blocks from the center of disks and the formation of pores. However, the equilibration of the micellar structure would be substantially restricted by the slow chain migration of the high-molecular-weight polymer blocks and the fact that the evolution of the P2VP domain might be kinetically prevented by the collapsed PS corona, leading to the immergence of a mixture of perforation disks with various pore sizes).

In a further experiment, IPA, which is a good solvent for the P2VP block, but a poor solvent for the PS and PB blocks, was employed to replace the role of acetone. However, across the full range of mixed IPA/cyclohexane solvents, PS_580_-*b*-PB_660_-*b*-P2VP_920_ exclusively self-assembled into spherical micelles, but failed to formed disk-like micelles (Fig. S8†). Again, it indicated that the combination of acetone and cyclohexane, which create a frustrating situation for the dissolution and coalescence of the PS, PB and P2VP blocks, is essential for the formation of the low-curvature micellar structures.

We also explored the self-assembly of PS_370_-*b*-PB_230_-*b*-P2VP_350_ and PS_260_-*b*-PB_170_-*b*-P2VP_300_ with much smaller molecular weights than PS_580_-*b*-PB_660_-*b*-P2VP_920_ in the acetone/cyclohexane mixtures. In an analogous manner, PS_370_-*b*-PB_230_-*b*-P2VP_350_ self-assembled into spherical micelles under low (≤30/70) and high (≥80/20) acetone/cyclohexane ratios, but formed complex micellar structures in the middle regions (40/60–70/30) (Fig. S9†). Irregular disk arrays were formed at acetone/cyclohexane = 50/50 and 60/40 (Fig. S9f and g†), while ill-shaped toroidal micelles emerged at acetone/cyclohexane = 65/35 (Fig. S9h†). Besides, pearl necklace-like structures were also observed upon a slightly higher feeding of acetone (Fig. S9i†). Similarly, PS_260_-*b*-PB_170_-*b*-P2VP_300_ self-assembled into spherical micelles in 30/70 and 70/30 acetone/cyclohexane mixtures, but formed slightly perforated disk-like micelles from acetone/cyclohexane = 40/60 to 60/40 (Fig. S10†). The height of the resulting disk-like micelles decreased from ∼6 to ∼3 nm, while the diameter expanded, with an increase in the acetone fraction (Fig. S10b–d and f†).

## Conclusions

In conclusion, we have demonstrated a facile method to prepare disk-like micelles through the solution self-assembly of PS-*b*-PB-*b*-P2VP triblock copolymers in the mixtures of acetone and cyclohexane. A diverse array of flat micellar structures, including disk arrays, disk clusters, uniform disks, and perforated disks, were observed upon tuning the volume ratio of the mixed solvents. The drastically distinct solvation and aggregation preference of the PS, PB and P2VP blocks in this frustrating solvent system may interpret the sophisticate self-assembly behaviour, although the mechanism clearly needs further investigation. The strategy developed in this work involving the combination of polar and nonpolar (near-)*Θ* solvents in the self-assembly of amphiphilic block copolymers provides an efficient pathway to low-curvature micellar structures. Future work is under process on the self-assembly of other PS-containing block copolymers, as well as the fabrication of patches on the disk-like micelles, utilizing cross-linking and post modification techniques.

## Conflicts of interest

There are no conflicts to declare.

## Supplementary Material

RA-009-C9RA01145E-s001
