# Supplementary material for: Formation of disk-like micelles of triblock copolymers in frustrating solvents
Source: RSC Adv. 2019 Mar 25;9(17):9443–8. doi: 10.1039/c9ra01145e (PMC9062166; doi:10.1039/c9ra01145e)
Supplement: RA-009-C9RA01145E-s001 [file RA-009-C9RA01145E-s001.pdf]

Electronic Supplementary Material (ESI) for RSC Advances.
This journal is © The Royal Society of Chemistry 2019

Electronic Supplementary Information

for

Formation of Disk-Like Micelles of Triblock Copolymers in Frustrating Solvents

Hongyan Zhu,[a,b] Yan Cui,[c] Jie Wang,*[a,d] and Huibin Qiu*[c]

[a]*Shanghai Institute of Applied Physics, Chinese Academy of Sciences, Shanghai 201800, China.*

[b]*University of Chinese Academy of Sciences, Beijing 100049, China.*

[c]*School of Chemistry and Chemical Engineering, State Key Lab of Metal Matrix Composites, Shanghai Jiao Tong University, Shanghai 200240, China*

[d]*Shanghai Advanced Research Institute, Zhangjiang Lab, Chinese Academy of Sciences, Shanghai 201204, China.*

*To whom correspondence should be addressed, E-mail: hbqiu@sjtu.edu.cn; wangjie@sinap.ac.cn;

Calculation of Flory-Huggins parameters between polymers and solvents

Hansen solubility parameters (δ) includes three contributions from dispersion forces (d), polar forces (p) and hydrogen-bonding (h) effects, namely δ_d, δ_p and δ_h, respectively, and is denoted as:

$$\delta_i^2 = \delta_{i,d}^2 + \delta_{i,p}^2 + \delta_{i,h}^2$$

The solubility parameters of polymers and solvents used in this work were listed in Table S1.[1,2]

The Flory-Huggins (FH) parameters (χ)[3] were calculated using Hansen solubility parameters based on the FH/Hansen model, and is defined as:

$$\chi_{12} = \alpha \frac{\upsilon_1}{RT}\left[(\delta_{1,d} - \delta_{2,d})^2 + 0.25(\delta_{1,p} - \delta_{2,p})^2 + 0.25(\delta_{1,h} - \delta_{2,h})^2\right]$$

Where the subscripts 1 and 2 denote the solvent and the polymer, respectively. R, T and υ are gas constant, temperature and molar volume, respectively. Moreover, the best value of α is 0.6.[1,3] Temperature is 25 °C.

If $\chi_{12} < 0.5$, a polymer and solvent are completely miscible.

The Flory-Huggins parameters between polymers and solvents were calculated and concluded in Table S2.

Table S1. Solubility parameters of polymers and solvents.

Solvents and polymers	Molar Volume (cm^3/mol)	Solubility parameters ($MPa^{1/2}$)			
		δ_d	δ_p	δ_h	δ_i
**Tetrahydrofuran**	81.7	16.8	5.7	8.0	19.4
**Acetone**	74	15.5	10.4	7.0	20.1
**Cyclohexane**	108.7	16.8	0	0.2	16.8
**Isopropanol**	76.8	15.8	6.1	16.4	23.5
**PS**	--	21.3	5.8	4.3	22.5
**PB**	--	17.0	0	1.0	17.0
**P2VP**	--	16.3	7.1	11.6	21.2

Table S2. Flory-Huggins parameters (χ) between polymers and solvents.

χ	PS	PB	P2VP
**Tetrahydrofuran**	0.46	0.40	0.08
**Acetone**	0.73	0.69	0.15
**Cyclohexane**	0.86	0.01	1.19
**Isopropanol**	1.24	1.30	0.12

Proton nuclear magnetic resonance (^{1}H NMR) analyses

All spectra used for characterization were obtained using deuterated chloroform ($CDCl_3$) as the solvent. The ^{1}H chemical shifts were determined using residual signals of $CDCl_3$ (7.26) as the internal standard, and were reported in parts per million (ppm). For PS-*b*-PB-*b*-P2VP, the aromatic peaks of PS is from 6.2 to 7.2 ppm, and the unsaturated functions of PB is from 4.8 to 5.7 ppm (including 5.2-5.7 ppm for poly(1,4-butadiene) and 4.8-5.2 ppm for poly(1,2-butadiene)), and the pyridine functions of P2VP is from 8.1 to 8.4 ppm. The peak positions of PS-*b*-PB-*b*-P2VP are marked on the corresponding NMR spectra with red-letters (**a, b, c, d**). (See Figures. S1, S3 and S5)

Calculation of block ratios

For PS-*b*-PB-*b*-P2VP, the absolute molar mass of the PS block was first determined by gel permeation chromatography (GPC). The absolute molecular weights of the other two blocks (PB and P2VP) were then determined by combining the molecular weight M_n of the PS block from GPC measurements with the block ratio of the triblock copolymer which was obtained by integration of the ^{1}H NMR spectrum. Taking PS_{580}-*b*-PB_{660}-*b*-$P2VP_{920}$ as an example:

The integration of **a** is assigned to 1.

The integration of **b** is 6.15, including the 5 aromatic hydrogens of PS and the rest 3 hydrogens of P2VP. Since the integration of **a** is assigned to 1, the integration of the rest 3 hydrogens of P2VP is 3. Thus, the integration of the 5 hydrogens of PS is 3.15.

The integration of **c** and **d** are 0.90 and 1.07, respectively. Integration of **c** represents 2 hydrogens of poly(1,4-butadiene) and one hydrogens of poly(1,2-butadiene). Integration of **d** represents 2 hydrogens of poly(1,2-butadiene). It concluded the following relationships:

$$\frac{2x+(n-x)}{2(n-x)}=\frac{0.90}{1.07}.$$

Thus, the proportion of poly(1,4-butadiene) is $x/n \approx 25.4\%$, and the proportion of poly(1,2-butadiene) is $(n-x)/n \approx 74.6\%$.

The number-average molecular weight of the PS block determined by GPC is 60400 g/mol, and the molecular weight of styrene is 104.15 g/mol. Thus, the degree of polymerization of PS is m = 60400/104.15 ≈ 580.

Combining the degree of polymerization of the PS block with the block ratio of PS and PB which is obtained by integration of the ^{1}H NMR spectrum. That is:

$$\frac{3.15}{1.97}=\frac{580\times 5}{(2\times 25.4\%+3\times 74.6\%)\times n}.$$

Thus, n ≈ 660.

Similarly, for PS and P2VP:

$$\frac{3.15}{1}=\frac{580\times 5}{k},$$

Thus, k ≈ 920.

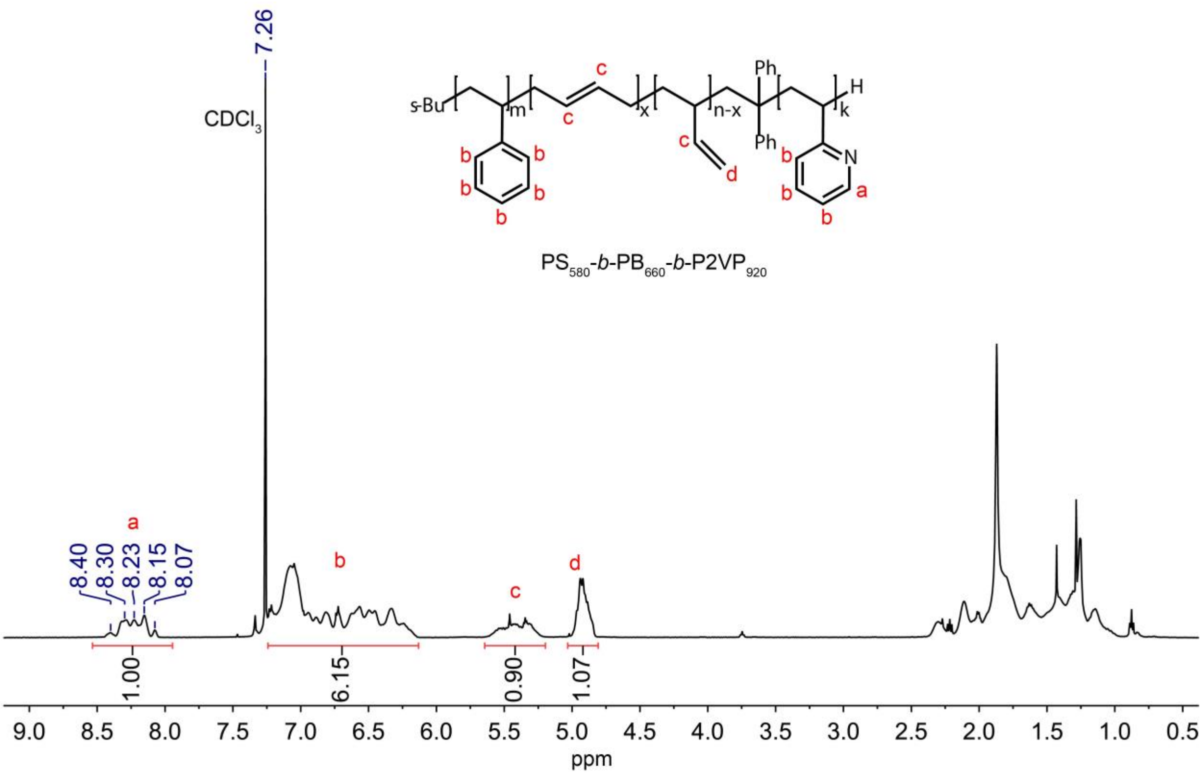

Figure S1. ^{1}H NMR spectrum of PS_{580}-*b*-PB_{660}-*b*-$P2VP_{920}$.

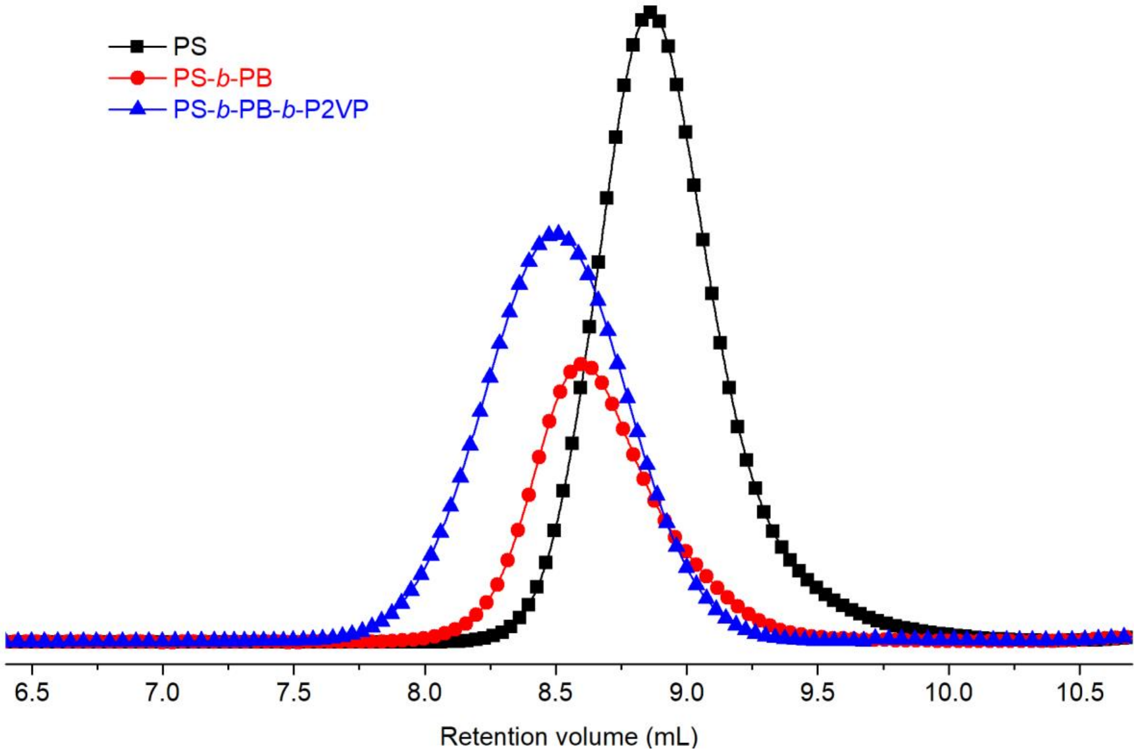

Figure S2. GPC traces of PS_{580}-*b*-PB_{660}-*b*-$P2VP_{920}$ and the PS homopolymer and PS-*b*-PB diblock copolymer intermediates.

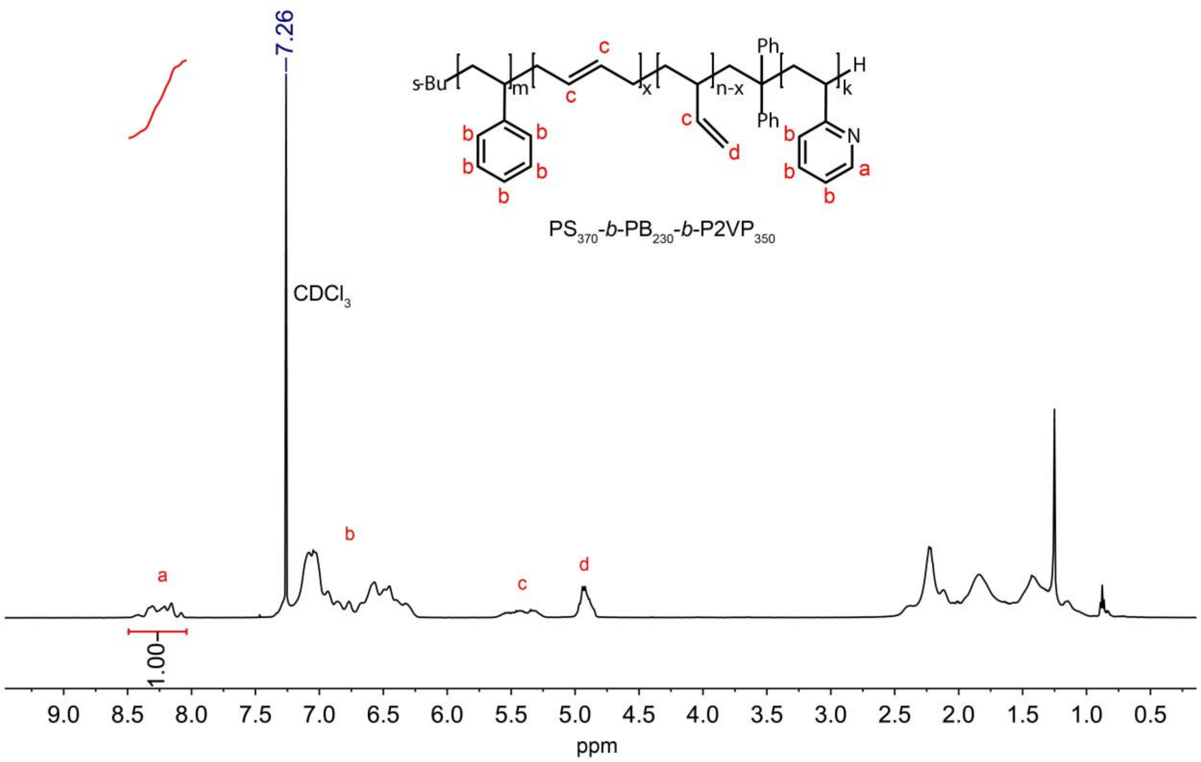

Figure S3. 1H NMR spectrum of PS_{370}-*b*-PB_{230}-*b*-$P2VP_{350}$.

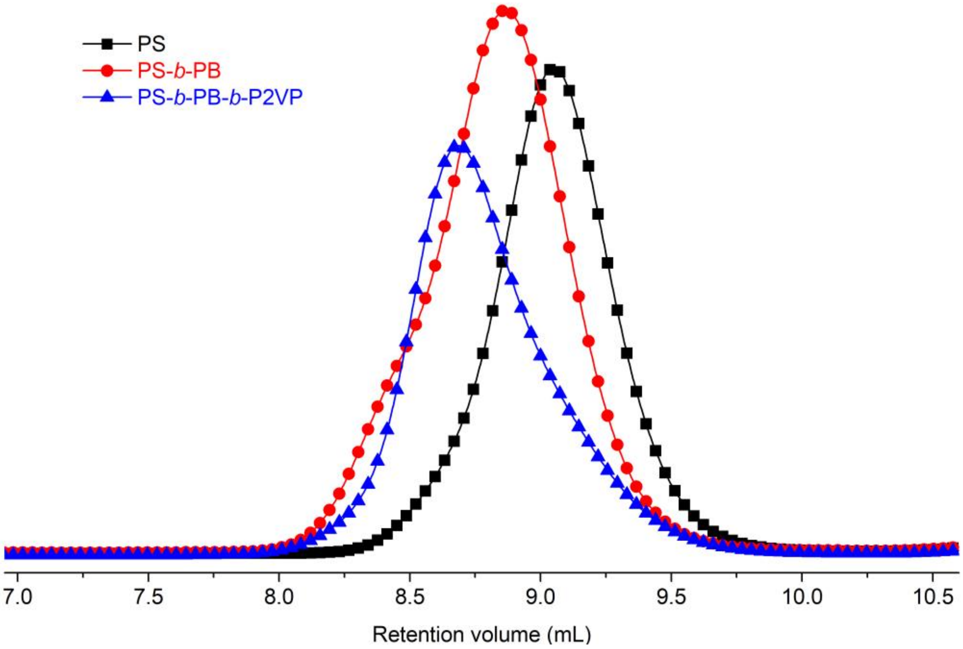

Figure S4. GPC traces of PS_{370}-*b*-PB_{230}-*b*-$P2VP_{350}$ and the PS homopolymer and PS-*b*-PB diblock copolymer intermediates.

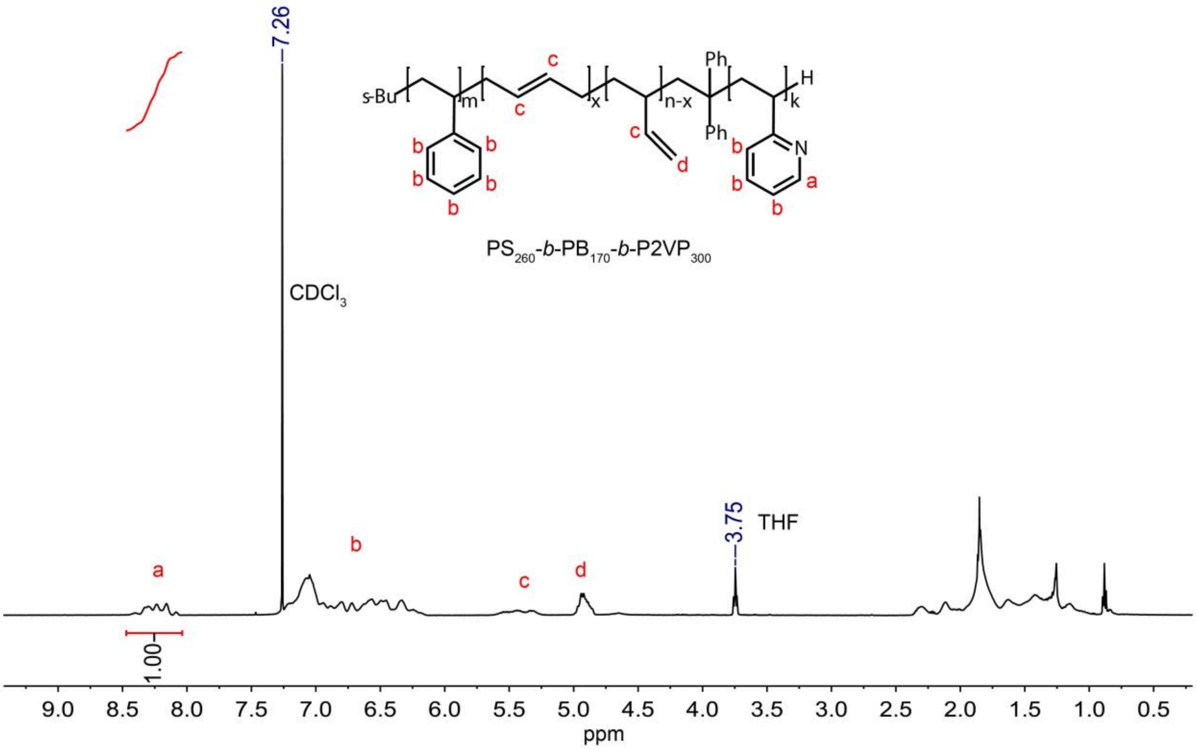

Figure S5. 1H NMR spectrum of PS_{260}-*b*-PB_{170}-*b*-$P2VP_{300}$.

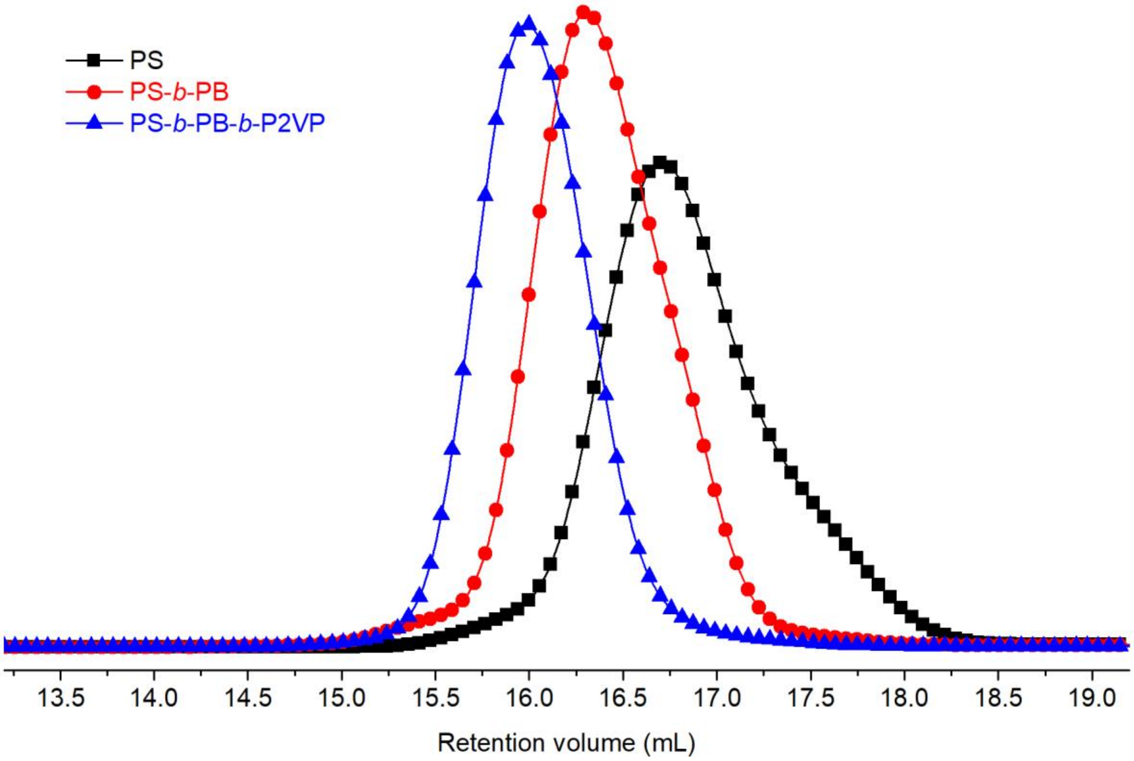

Figure S6. GPC traces of PS_{260}-*b*-PB_{170}-*b*-$P2VP_{300}$ and the PS homopolymer and PS-*b*-PB diblock copolymer intermediates.

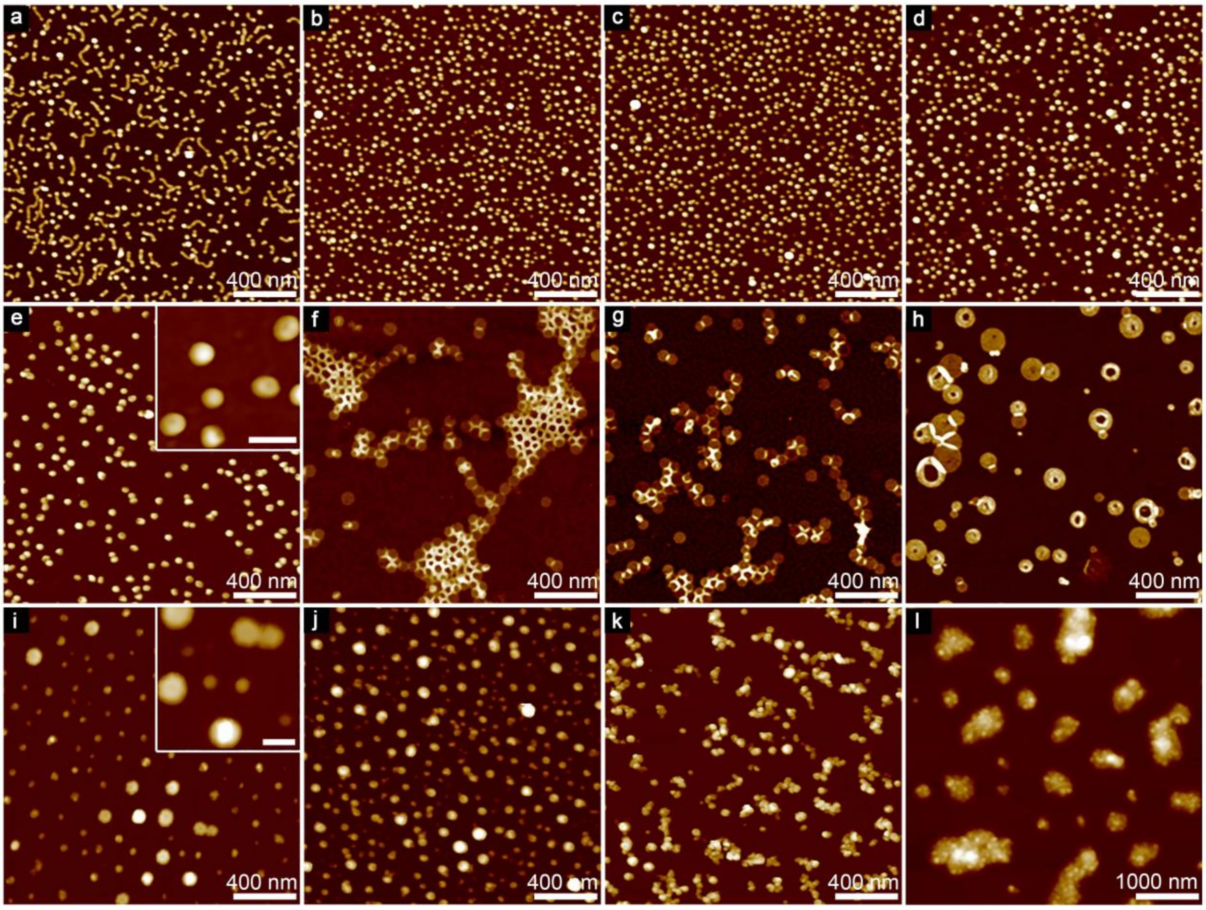

Figure S7. AFM height images of various micellar structures formed by the self-assembly of PS_{580}-*b*-PB_{660}-*b*-$P2VP_{920}$ in different acetone/cyclohexane mixtures with acetone to cyclohexane volume ratios of (a) 0/100, (b) 10/90, (c) 20/80, (d) 30/70, (e) 40/60, (f) 45/55, (g) 50/50, (h) 65/35, (i) 70/30, (j) 80/20, (k) 90/10, (l) 100/0 (v/v). The scale bars correspond to 100 nm in the inset images.

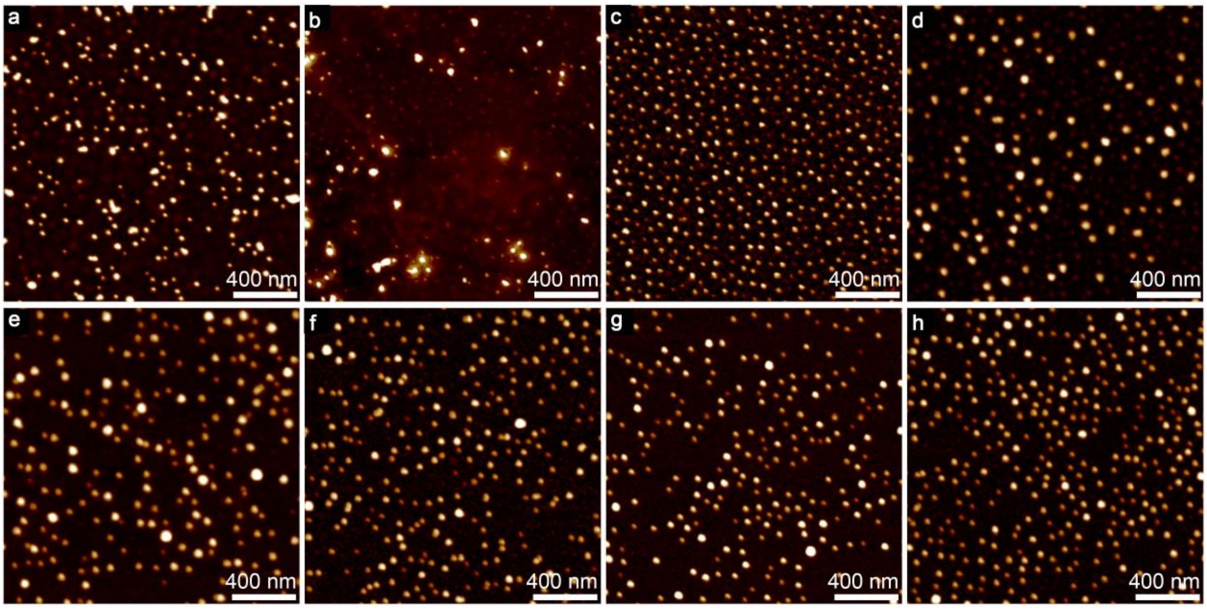

Figure S8. AFM height images of various micellar structures formed by the self-assembly of PS_{580}-*b*-PB_{660}-*b*-$P2VP_{920}$ in different isopropanol/cyclohexane mixtures with isopropanol to cyclohexane volume ratios of (a) 10/90, (b) 20/80, (c) 30/70, (d) 40/60, (e) 50/50, (f) 60/40, (g) 70/30, (h) 80/20 (v/v).

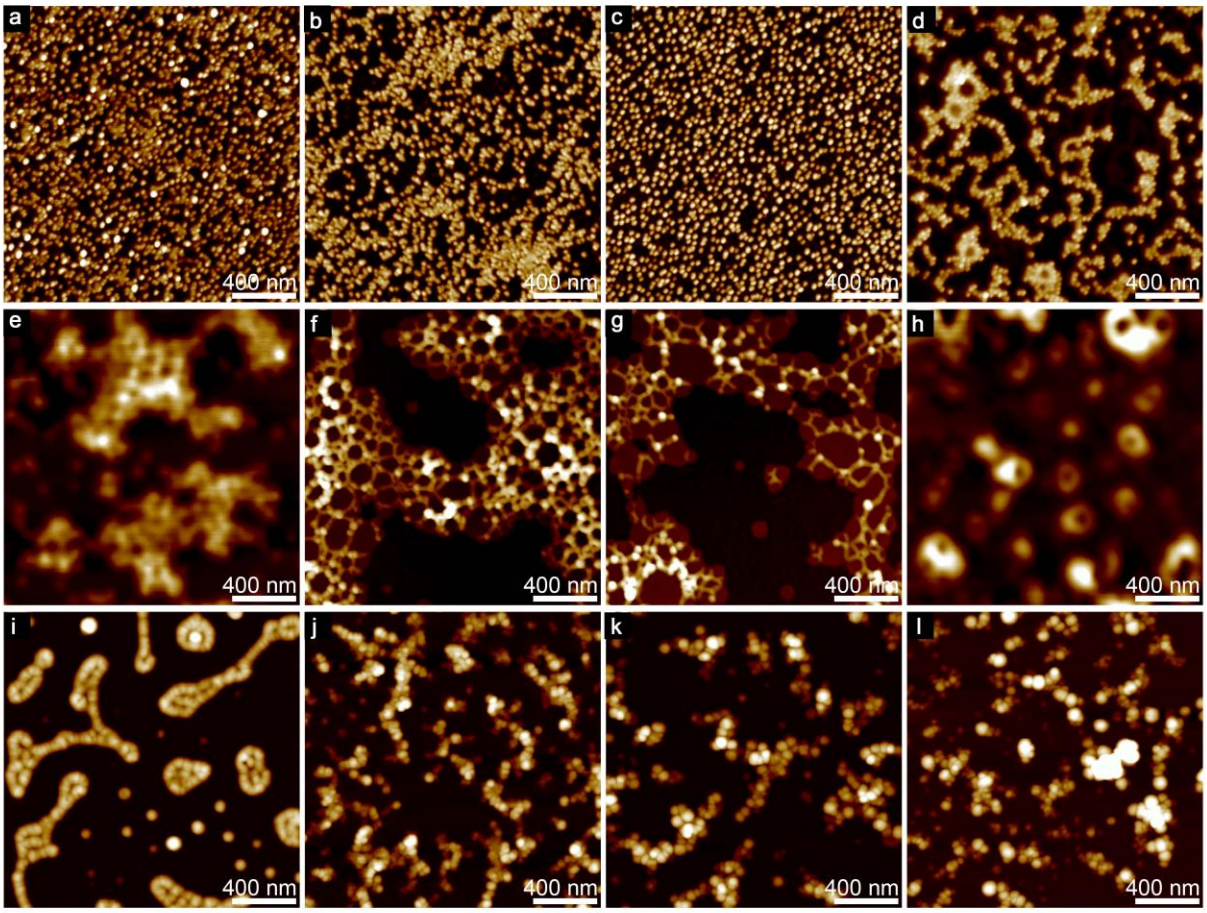

Figure S9. AFM height images of various micellar structures formed by the self-assembly of PS_{370}-*b*-PB_{230}-*b*-$P2VP_{350}$ in different acetone/cyclohexane mixtures with acetone to cyclohexane volume ratios of (a) 0/100, (b) 10/90, (c) 20/80, (d) 30/70, (e) 40/60, (f) 50/50, (g) 60/40, (h) 65/35, (i) 70/30, (j) 80/20, (k) 90/10, (l) 100/0 (v/v).

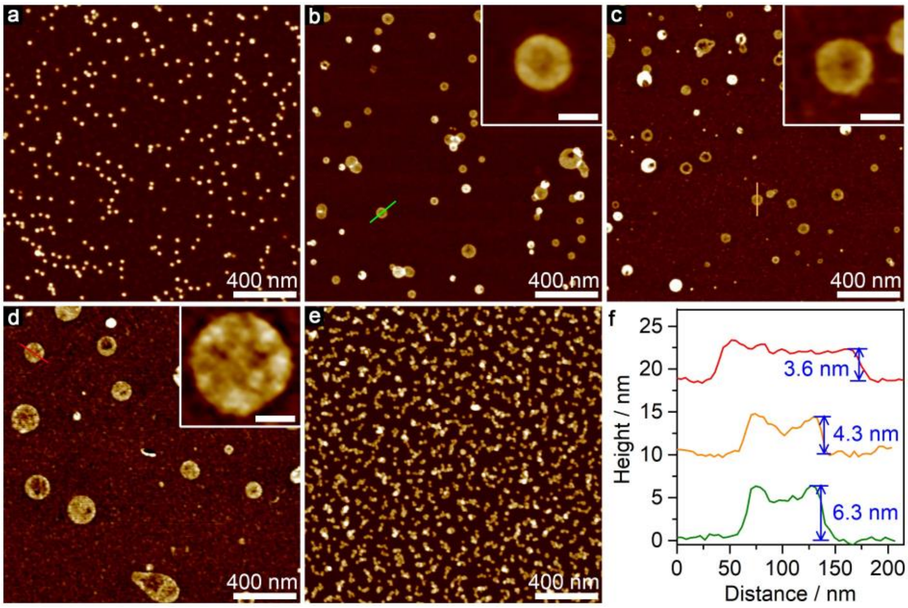

Figure S10. AFM height images of various micellar structures formed by the self-assembly of PS_{260}-*b*-PB_{170}-*b*-$P2VP_{300}$ in different acetone/cyclohexane mixtures with acetone to cyclohexane volume ratios of (a) 30/70, (b) 40/60, (c) 50/50, (d) 60/40, (e) 70/30 (v/v). (f) Height profiles of the disk-like structures shown in (b-d). The scale bars correspond to 50 nm in the inset images.

1 C. M. Hansen, *Hansen solubility parameters: a user's handbook*, CRC press, 2002.
2 M. M. Arras, B. He and K. D. Jandt, *Polymer*, 2017, **127**, 15-27.
3 T. Lindvig, M. L. Michelsen and G. M. Kontogeorgis, *Fluid Phase Equilib.*, 2002, **203**, 247-260.
